# The “Intubox”: Enhancing Frontline Healthcare Worker Safety During Coronavirus Disease 2019 (COVID-19)

**DOI:** 10.7759/cureus.8530

**Published:** 2020-06-09

**Authors:** Feroza Motara, Abdullah E Laher, Jana Du Plessis, Muhammed Moolla

**Affiliations:** 1 Emergency Medicine, University of the Witwatersrand, Johannesburg, ZAF

**Keywords:** covid-19, sars-cov-2, aerosol box, intubox, intubation, healthcare worker safety, frontline staff, mechanical ventilation, emergency medicine, critical care

## Abstract

There has been a substantial burden of healthcare worker infection during the current coronavirus (COVID-19) pandemic, likely due to a lack of adequate preparedness, suboptimal institutional infection control measures, atypical patient presentation, poor compliance with personal protective equipment (PPE) and exposure to high-risk aerosol generating procedures, such as endotracheal intubation. There is significant concern that developing countries will face heightened levels of staff exposure during the COVID-19 pandemic. To mitigate this exposure risk during procedures, such as endotracheal intubation, various “aerosol boxes” have been designed by frontline healthcare workers. However, in practice these boxes were found to hamper endotracheal intubation and other procedures due to the limited space and manoeuvrability they allow. To further reduce particle dispersion and to improve on the practicality and ergonomic design of the prototype “aerosol box”, the Intubox was developed by staff at the Charlotte Maxeke Johannesburg Academic Hospital after instituting several changes to the prototype design.

## Introduction

Severe acute respiratory syndrome coronavirus 2 (SARS-COV-2) disease coronavirus (COVID-19) was first reported in Wuhan, China at the end of 2019. The virus has since spread across the globe, affecting 213 countries and territories with over six million confirmed cases and close to 400,000 reported deaths as at the end of May 2020 [1]. Similar to previous viral disease outbreaks and epidemics, there has been a substantial burden of healthcare worker infection [2]. Probable reasons include a lack of adequate preparedness, suboptimal institutional infection control measures, poor compliance with personal protective equipment (PPE) and exposure to high-risk aerosol generating procedures, such as endotracheal intubation, airway suctioning and nebulization [3].

Morbidity and mortality among healthcare workers infected with COVID-19 are suspected to be higher than that of the general population, possibly due to exposure to a higher viral burden during patient care [4]. In addition, the relatively higher estimated basic reproductive number (R_0_ ≈ 2-3) associated with SARS-CoV-2, exposure to asymptomatic carriers presenting with non-COVID-19 related illnesses and unusual patient presentations, such as strokes, Guillain-Barre syndrome, cardiovascular complications and skin rashes, further compound the risk facing frontline healthcare workers [5,6].

There is significant concern that developing countries will face heightened levels of staff exposure during the COVID-19 pandemic. Fragile health systems and a large burden of chronic diseases, such as HIV and tuberculosis in African countries, coupled with limited resources of PPE and advanced critical care facilities will likely have an adverse effect on the ability to respond to the COVID-19 pandemic [7]. South Africa currently has the highest number of confirmed COVID-19 cases on the continent of Africa, highlighting the need for disaster preparedness and accelerated preparation for a potential surge in critically ill patients [1]. 

To mitigate this exposure risk during procedures, such as endotracheal intubation, various “aerosol boxes” have been designed by frontline healthcare workers [8]. The majority of these boxes consist of a simple three-sided Perspex box with two armholes through which the patient airway can be accessed. In a simulation mannequin-based study where fluorescent dye and ultraviolet light were used to track dispersion of droplets and aerosol particles after a simulated cough, the “aerosol box” was shown to substantially reduce clinician exposure to aerosol particles [9]. However, in practice these boxes were found to hamper endotracheal intubation and other procedures due to the limited space and maneuverability they allow [10]. 

## Technical report

**Figure 1:**
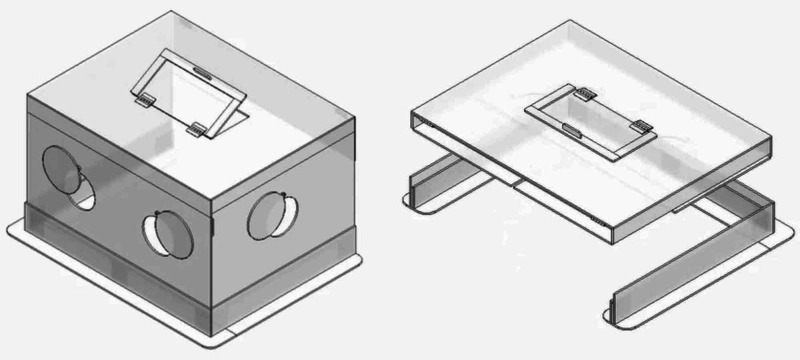
The “Intubox”. Left: upright and ready for use. Right: collapsible for transport and storage.

To further reduce particle dispersion and to improve on the practicality and ergonomic design of the prototype “aerosol box”, staff at the Charlotte Maxeke Johannesburg Academic Hospital (CMJAH) emergency department (ED) designed and developed the Intubox (Figure 1, Video 1) after instituting several changes to the prototype design. CMJAH is a 1068-bed tertiary care academic hospital in Johannesburg, South Africa, employing approximately 4,500 staff members. Approximately 3,500 patients attend the ED monthly, with roughly a third requiring admission. 

**Video 1:** The Intubox.

Changes that were instituted to the prototype design included the following:

1. Movable flaps were added to the two arm ports located at the proximal end of the box.

2. To accommodate for easy passage of essential items, such as a suction catheter, and to facilitate assistance with procedures, such as bag-valve-mask ventilation, an arm port with a cover flap was added to the right-side panel of the box.

3. To facilitate removal of the endotracheal tube introducer and delivery of other rescue devices, such as a bougie or laryngeal mask airway, an additional port with a cover flap, opening away from the laryngoscopist, was added to the upper panel of the box.

4. To accommodate larger patients, a hinged flap was added to the distal top end of the box.

5. To allow collapsibility of the box for easy storage and transport, multiple hinges were added.

6. To further reduce particle dispersion and to create an adjustable seal over the patient’s thorax, a disposable transparent plastic sheet can be taped down over the distal open end of the box.

## Discussion

The Intubox has already been successfully used on multiple patients requiring intubation and post-intubation care. It has also been left in situ for the entire duration of patient ventilation so as to reduce particle dispersion during procedures, such as airway suctioning (when closed suctioning is not available). Due to the long half-life of viral particles on surfaces, this may further reduce healthcare worker exposure in the aftermath of aerosol generating procedures [11]. The box could potentially also be adapted for use in non-ventilated patients being managed with face-mask oxygen, high-flow nasal oxygen or non-invasive ventilation. Prior to reusing the Intubox on another patient, it must be sterilized using a hypochlorite solution in a concentration of 1,000 ppm while donning appropriate PPE.

Supine positioning of the patient, patient cooperation, availability of an experienced laryngoscopist and availability of a video laryngoscope are the prerequisites that may limit the use of the Intubox. Furthermore, the Intubox does not obviate the need for appropriate healthcare worker PPE.

## Conclusions

It is anticipated that the Intubox will play a significant role in improving frontline healthcare worker safety, both in South Africa and abroad. It is hoped that by creating an additional barrier layer between the suspected COVID-19 positive patient and healthcare workers and by containing infective droplet spread, the risk of infection to staff and other patients may be significantly mitigated.
